# A meta-analysis on the risk of esophageal cancer in type 2 diabetes patients treated with GLP-1 receptor agonists

**DOI:** 10.3389/fendo.2025.1532587

**Published:** 2025-03-20

**Authors:** Qi Wu, Yan Zeng, Yong Liu, Fangyuan Teng, Tiejun Zhou, Man Guo, Zongzhe Jiang, Yong Xu

**Affiliations:** ^1^ Department of Pathology, The Affiliated Hospital of Southwest Medical University, Luzhou, Sichuan, China; ^2^ Department of Endocrinology and Metabolism, The Affiliated Hospital of Southwest Medical University, Luzhou, Sichuan, China; ^3^ Metabolic Vascular Disease Key Laboratory of Sichuan Province, and Sichuan-Chongqing Joint Key Laboratory of Metabolic Vascular Diseases, Luzhou, Sichuan, China; ^4^ Precision Pathology Diagnosis for Serious Diseases Key Laboratory of LuZhou, Luzhou, Sichuan, China; ^5^ Department of Intensive Care Unit, The Affiliated Hospital of Southwest Medical University, Luzhou, China; ^6^ Department of Intensive Care Unit, Wangcang People’s Hospital, Guangyuan, China; ^7^ Dr. Neher’s Biophysics Laboratory for Innovative Drug Discovery, State Key Laboratory of Quality Research in Chinese Medicine, Faculty of Chinese Medicine, Macau University of Science and Technology, Macao SAR, China

**Keywords:** glucagon-like peptide-1 receptor agonist, semaglutide, type 2 diabetes mellitus, esophageal cancer, meta-analysis

## Abstract

**Objective:**

To evaluate the association between glucagon-like peptide-1 receptor agonists (GLP-1 RAs) treatment and the risk of esophageal cancer in adults with type 2 diabetes mellitus (T2DM) or obesity through a comprehensive meta-analysis.

**Methods:**

A systematic computerized searches and collection of eligible randomized controlled trials (RCTs) was performed to compare the risk of esophageal cancer between GLP-1 RA and control agents. The bias risks and quality of the studies were evaluated, and a meta-analysis was conducted using Stata 18.0 and R 4.0.2 statistical software.

**Results:**

The meta-analysis included data from six studies involving 13,391 participants. The pooled relative risk (RR) of esophageal cancer in patients using GLP-1 RAs compared to control agents was 0.46 (95% CI 0.13-1.59; p=0.725; I²=0%). Subgroup analyses stratified by age groups, intervention durations, BMI categories, and indications for T2DM or obesity treatment more often indicated no association between GLP-1 RAs use and increased risk of esophageal cancer.

**Conclusions:**

GLP-1 RAs did not increase the incidence of esophageal neoplasms, and there were not probably significant within-class differences in T2DM or obesity treatment. This finding supports the safety of GLP-1 RAs as a therapeutic option for the clinical management of T2DM.

**Systematic review registration:**

https://www.crd.york.ac.uk/PROSPERO/, identifier CRD42024543945.

## Introduction

Glucagon-like peptide-1 receptor agonists (GLP-1 RAs) are a class of novel antidiabetic agents that primarily exert their glucose-lowering effects by stimulating insulin secretion, inhibiting glucagon release, and promoting satiety. GLP-1 RAs have been shown to significantly reduce blood glucose levels and body weight in patients with type 2 diabetes mellitus (T2DM) (1). Additionally, certain GLP-1 RAs offer established cardiovascular protection and may provide potential renal benefits (2). As a result, the therapeutic role of GLP-1 RAs has been increasingly emphasized in both national and international clinical guidelines (3). However, between 40% and 70% of patients experience gastrointestinal side effects such as nausea, vomiting, diarrhea, or constipation during treatment with GLP-1 RAs. Research suggests that the use of GLP-1 RAs for weight loss may elevate the risk of pancreatitis, gastroparesis, and intestinal obstruction (4). Furthermore, patients with T2DM receiving GLP-1 RAs therapy are at an increased risk of developing gastroesophageal reflux, esophageal strictures, and Barrett’s esophagus. Some studies have indicated a potential association between GLP-1 RAs use and an increased incidence of esophageal cancer (5). Conversely, a retrospective cohort study found that GLP-1 RAs might exert a protective effect against esophageal cancer (6). Patients with T2DM, especially those with poorly controlled blood glucose levels (glycated hemoglobin, HbA1c, ≥ 7.0%) and coexisting inflammatory conditions, are at a higher risk of developing malignancies (7). Currently, the precise relationship between GLP-1 RAs use and the incidence of esophageal cancer remains unclear.

In summary, the impact of different types and doses of GLP-1 receptor agonists on esophageal cancer incidence in patients with type 2 diabetes or obesity has not been fully elucidated. Therefore, we conducted the first meta-analysis aimed at evaluating the association between GLP-1 RAs treatment and the risk of esophageal cancer. Additionally, we performed subgroup analyses based on variables such as dosage, duration of treatment, treatment indication (type 2 diabetes or weight loss), mean age, and mean body mass index (BMI) to assess the influence of these confounding factors on the study outcomes. By conducting a quantitative analysis, we aim to provide evidence-based support for the clinical use of GLP-1 RAs.

## Methods

### Search strategy

The protocol for this meta-analysis has been registered in PROSPERO (CRD42024543945). This study was conducted in accordance with the Preferred Reporting Items for Systematic Reviews and Meta-Analyses (PRISMA) 2020 guidelines (8). We performed a comprehensive search of the PubMed, Embase, Web of Science, Scopus, and Cochrane Central Register of Controlled Trials (CENTRAL) databases, from their inception until June 13, 2024. Additionally, we searched for unpublished and ongoing trials in ClinicalTrials.gov, manually reviewed the reference lists of relevant systematic reviews and meta-analyses, and examined grey literature available in clinical trial registries, without language restrictions. The search strategy included the following terms: (glucagon like peptide-1 receptor agonists OR GLP-1 receptor agonist OR albiglutide OR dulaglutide OR exenatide OR liraglutide OR lixisenatide OR semaglutide OR tirzepatide) AND (esophageal neoplasms) AND (randomized controlled trial), see **Appendix 1** in **Supplementary Data Sheet 1** for details.

### Eligibility criteria

We included clinical trials based on the following criteria: (1) participants were adults aged 18 or 20 years and older with type 2 diabetes or obesity (2) who received GLP-1 RAs, either as monotherapy or adjunct therapy (3) at least 52 weeks. (4) Studies reported the incidence of esophageal cancer (5) for randomized controlled trials (RCTs) were included. We excluded studies that comparing different types, frequencies, or doses of GLP-1 receptor agonists, with no available data nor non-comparative. Reviews, opinion articles, editorials, case reports, conference abstracts, and expert opinions are also excluded (**Appendix 3** in **Supplementary Data Sheet 1**).

### Data extraction and quality assessments

Data were independently extracted by two authors (Qi Wu and Yan Zeng) and subsequently reviewed and arbitrated by a third examiner (Man Guo) using a pre-specified data extraction form to ensure accuracy. The following information was extracted from each eligible study: study characteristics (study name, first author’s name, publication year, and country), participant demographics (sample size, age, duration of diabetes or obesity, BMI, fasting blood glucose (FPG), and HbA1c, and intervention details (name, frequency, and dosage, see **Appendix 5** in **Supplementary Data Sheet 1** for details). For the incidence of esophageal cancer, the total number of participants in both the experimental and control groups was estimated. If multiple publications were available for the same study population, the most recent and comprehensive report was included, with the longest follow-up time and the largest set of primary data. All continuous variables were rounded to one decimal place.

### Bias risk assessment

We used the Cochrane Risk of Bias 2 (RoB 2) tool to assess the risk of bias in the included RCTs, evaluating six specific domains: sequence generation, allocation concealment, blinding, incomplete outcome data, selective outcome reporting, and other potential sources of bias (https://methods.cochrane.org/risk-bias-2). Qi Wu independently assessed the risk of bias and calculated detailed descriptions of all studies.

### Data synthesis and statistical analysis

A DerSimonian-Laird random-effects model was used to analyze heterogeneous data in the meta-analysis. The effect measure was expressed as the relative risk (RR) of esophageal cancer incidence, along with its 95% confidence intervals (CIs). Heterogeneity across studies was assessed using Cochrane’s Q statistic (χ² test), the I² statistic, and visual inspection of forest plots. Statistical heterogeneity was considered significant when p < 0.05 or I² > 50%. Subgroup analyses were performed based on baseline mean age (≤60 years or >60 years), BMI, treatment duration of GLP-1 RAs (≤52 weeks or >52 weeks), treatment indication (T2DM or obesity), and the type of comparator (placebo or other antidiabetic drugs). Publication bias was evaluated visually using funnel plots and quantitatively with Egger’s test. All statistical analyses were conducted using Stata 18.0 (StataCorp, College Station, TX) and R version 4.0.2.

## Results

### Trial identification and characteristics

**Figure 1:**
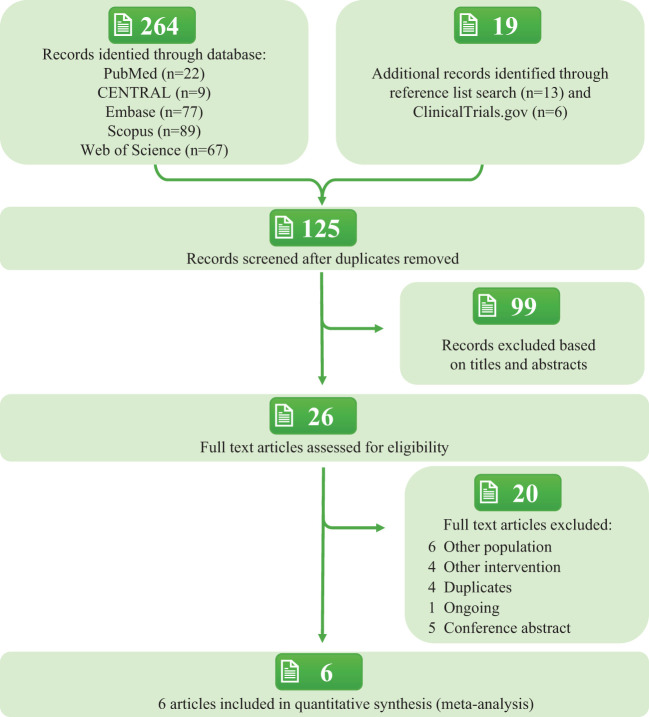
PRISMA flow diagram.

Based on the search strategy, 283 articles were initially identified, and 26 full-text articles were evaluated. Of these, 6 articles (5, 9–13) involving 5 RCTs met the inclusion criteria for the meta-analysis (**Figure 1**). The included RCTs involved a total of 13,391 participants, with intervention durations ranging from 52 to 156 weeks. The mean age (standard deviation, SD) was 62.4 (9.9) years, body weight was 89.1 (20.8) kg, BMI was 32.2 (6.0) kg/m², FPG was 8.3 (2.1) mmol/L, and HbA1c was 8.5(1.6) %. The proportion of male participants was 64.3%. Detailed participant characteristics are presented in **Table 1** and **Appendix 4** in **Supplementary Data Sheet 1**. The quality assessment of the included studies is shown in **Appendix 6** in **Supplementary Data Sheet 1** and **Supplementary eFigure 1**. Most of the studies clearly described protocols such as randomization and double blinding.

**Table 1:** Baseline characteristics of randomized controlled clinical trials and participants included.

Study	Cohort name or NCT	Country and region	Population description	No. of participants	Intervention duration	Age (mean, SD), years	No. (%) of male	Wight (mean, SD), kg	BMI (mean, SD), kg/m^2^	FPG (mean, SD), (mmol/l)	HbA1c (mean, SD), %	Interventions	Controls	Overall risk of bias
**Sponsor: GlaxoSmithKline** (9)	NCT02465515 (Harmony Outcomes)	28 countries	T2DM	9463	140 weeks	64.1 (8.7)	6569 (69.4)	NR	32.3 (5.9)	NR	NR HbA1c 8.7 (1.5)	Albiglutide 30 mg QW+glargine; Albiglutide 50 mg QW+glargine	lispro + glargine	Low risk
**M. Diamant, 2012** (10)	NCT00641056 (DURATION-3)	the USA (and Puerto Rico), the European Union, Russia, Australia, Korea, Taiwan, and Mexico.	T2DM	456	84 weeks	58.0 (10.0)	243 (53.0)	90.9 (17.5)	32.0 (5.0)	9.8 (2.6)	8.3 (1.1)	Exenatide 2 mg QW	Insulin glargine: 10 IU/day	Some concerns
**M. Diamant, 2014** (11)	NCT00641056 (DURATION-3)	the USA (and Puerto Rico), the European Union, Russia, Australia, Korea, Taiwan, and Mexico.	T2DM	456	156 weeks	58.0 (10.0)	243 (53.0)	90.9 (17.5)	32.0 (5.0)	9.8 (2.6)	8.3 (1.1)	Exenatide 2 mg QW	Insulin glargine: 10 IU/day	Some concerns
**K. Kaku, 2018** (12)	NCT02207374	Japanese	T2DM	601	56 weeks	58.5 (10.3)	430 (71.5)	71.5 (15.5)	26.4 (4.7)	NR	8.1 (0.9)	Semaglutide 0.5 mg QW; Semaglutide 1.0 mg QW;	additional OAD	Low risk
**M. Kellerer, 2022** (5)	NCT03689374 (SUSTAIN 11)	21 countries	T2DM	1748	52 weeks	61.2 (9.5)	894 (51.1)	87.9 (18.3)	31.5 (5.5)	8.6 (0.7)	NR	Semaglutide 1.0 mg QW	insulin TID	Low risk
**F. K. Knop, 2023** (13)	NCT05035095 (OASIS 1)	9 countries across Asia, Europe, and North America	overweight or obesity	667	68 weeks	50.0 (13.0)	182 (27.0)	105.4 (22.2)	37.5 (6.5)	5.5 (0.6)	5.6 (0.3)	Semaglutide 50 mg QD	placebo plus lifestyle intervention	Low risk

Round the data to one decimal place. NCT, National Clinical Trial; NO., number of total participants; BMI, body mass index; FPG, fasting plasma glucose; HbA1c, glycosylated hemoglobin; T2DM, type 2 diabetes mellitus; NR, not report; QD, once daily; QW, once weekly.

### Esophageal cancer risk and GLP-1 RAs

**Figure 2:**
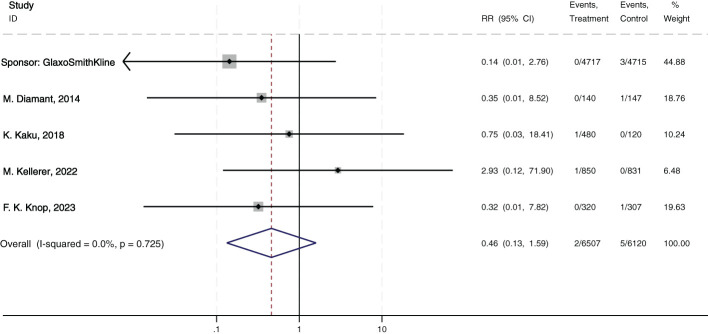
Risks of esophageal neoplasms in patients with GLP-1 RAs in all trials.

Neither albiglutide, exenatide, nor semaglutide was associated with an increased risk of esophageal cancer in patients with T2DM, with a pooled RR of 0.46 (95% CI: 0.13-1.59; p = 0.725; I² = 0%, **Figure 2**). This lack of association persisted irrespective of whether the drugs were used for diabetes or obesity management, as well as whether semaglutide was administered subcutaneously or orally (see **Supplementary eFigure 2** for details).

### Subgroup analysis

**Figure 3:**
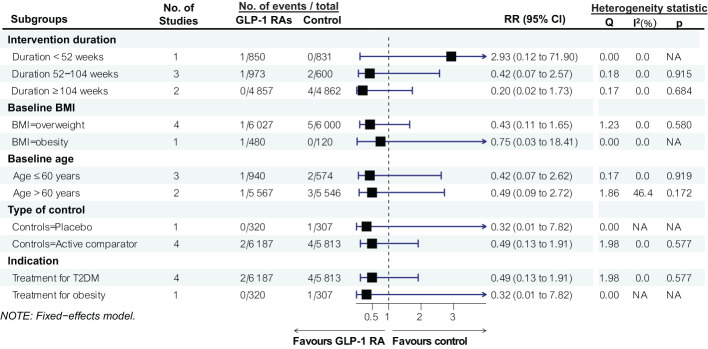
Factors and risks of esophageal neoplasms in RCTs of GLP-1 RA Drug Use.

Subgroup analyses stratified by baseline mean age (≤60 or >60 years), BMI categories (overweight: BMI 25 ≤ BMI < 30; obesity: BMI ≥ 30), duration of GLP-1 RAs treatment (≤52 weeks, 52–104 weeks, or >104 weeks), treatment indication (type 2 diabetes or obesity), and type of comparator (placebo or other antidiabetic drugs) maybe reveal no significant association between GLP-1 RAs and the risk of esophageal cancer in any of these subgroups (**Figure 3**).

### Publication bias

Egger’s test showed no evidence of publication bias (p = 0.224, t = 0.62). The funnel plot appeared largely symmetrical, further suggesting an absence of significant publication bias.

## Discussion

This meta-analysis demonstrates that treatment with GLP-1 RAs does not increase the risk of esophageal cancer, highlighting the safety of these drugs in this regard. The findings maybe hold true regardless of the intervention duration, treatment indication, or baseline characteristics of the patients (e.g., duration of T2DM or obesity, age, and BMI). To date, this study is the first and most comprehensive systematic review and meta-analysis assessing the safety of GLP-1 RA treatment with respect to esophageal cancer risk in patients with T2DM or obesity. Moreover, our research includes a larger sample size, broader coverage, and more detailed subgroup analyses. These findings offer significant statistical power to inform clinical practice and provide new insights for future research.

A real-world study queried the US Collaborative Network (comprising 63 healthcare organizations) within the TriNetX research database. After propensity score matching (PSM), 146,277 patients with T2DM aged ≥18 years were identified. Compared to non-users, patients treated with GLP-1 RAs exhibited a statistically significant lower risk of esophageal cancer (0.04% vs. 0.13%, p < 0.0001) at the seven-year follow-up mark (14). Another retrospective cohort study, utilizing a nationwide multicenter database of electronic health records (EHRs), included 1,651,452 patients with T2D prescribed GLP-1 RAs (mean [SD] age: 59.8 [15.1] years). This study found that GLP-1 RAs, compared to insulin, were associated with a significant reduction in esophageal cancer risk (HR, 0.60; 95% CI, 0.42–0.86) (6). However, these findings have limitations, including potential misdiagnosis, uncontrolled confounders, and the inability to account for modifiable risk factors such as diet and physical activity due to the retrospective nature of the studies. Additionally, as the database is U.S.-based, the generalizability of these findings to non-U.S. populations may be limited.

This study has several limitations. First, we were unable to include newer GLP-1 receptor agonists (such as orforglipron, retatrutide, and loxenatide) in our pooled analysis, due to constraints in the original clinical trials. Second, the primary endpoints of most published trials focused on metabolic parameters rather than esophageal cancer, which may introduce publication bias and potentially affect the accuracy of the results. Finally, the follow-up duration in the included RCTs may be insufficient to fully capture the long-term effects of GLP-1 RAs on esophageal cancer risk.

GLP-1 RAs regulate blood glucose and control body weight through various mechanisms, including stimulating insulin secretion and synthesis, inhibiting glucagon secretion, delaying gastric emptying, increasing satiety, and reducing appetite, thereby lowering caloric intake. Short-acting GLP-1 RAs, such as subcutaneous exenatide and lixisenatide, are particularly effective in controlling postprandial blood glucose levels due to their pronounced effects on gastric emptying and glucagon suppression (15). Long-acting GLP-1 RAs, such as albiglutide, dulaglutide, liraglutide, and semaglutide, are more effective in reducing HbA1c, fasting plasma glucose, and body weight, whether used in combination with basal insulin or alongside oral antidiabetic agents (16). The first large-scale study examining the relationship between GLP-1 RAs and the incidence of gastroesophageal reflux disease (GERD) and its complications, involving 127 million adult patients with T2DM, revealed an increased risk of first-time erosive esophagitis, Barrett’s esophagus, and non-erosive reflux disease, regardless of the GLP-1 RA type used. This may be associated with delayed gastric emptying. Shorter-acting GLP-1 RAs tend to delay gastric emptying more significantly than long-acting GLP-1 RAs, and long-acting agents generally do not increase the risk of long-term GERD-related outcomes (15). Additionally, a study conducted by researchers from the University of British Columbia, analyzing data from 16 million patients, found that the use of GLP-1 RAs was associated with an increased risk of gastroparesis and esophageal strictures (4). While it is well-established that esophageal cancer incidence increases with advancing age, this trend was not observed in our pooled analysis. This deviation may potentially be attributed to the use of GLP-1 RAs, suggesting a possible protective effect against esophageal cancer. However, this hypothesis remains speculative and requires future large-scale, multicenter, long-term follow-up clinical trials and epidemiological studies.

While GLP-1 RAs have been associated with GERD and esophageal complications such as erosive esophagitis and Barrett’s esophagus, retrospective cohort studies have also indicated that GLP-1 RAs may reduce the risk of esophageal cancer in patients with T2DM (6). However, our study found that GLP-1 RAs use does not increase the risk of esophageal cancer in patients with T2DM or obesity, supporting their use in clinical practice. However, clinical decision-making should also take into account other factors, such as gastrointestinal side effects. Therefore, the use of GLP-1 RAs for glycemic control or weight management should be carefully evaluated based on the patient’s individual circumstances and overall clinical profile.

The mechanisms underlying this protective effect may involve several factors: (1) Anti-inflammatory Properties: GLP-1 RAs exhibit potent anti-inflammatory effects, which could help reduce chronic local inflammation in the esophagus by inhibiting the release of pro-inflammatory mediators and mitigating oxidative stress. This anti-inflammatory action might potentially prevent the progression of inflammation into cancer (17). (2) Improvement of Metabolic Parameters: GLP-1 RAs are known to effective in controlling blood glucose and reducing body weight, both of which are established risk factors for esophageal cancer (18). By improving these metabolic parameters, GLP-1 RAs reduce the concentrations of insulin-like growth factor (IGF) binding protein, resulting in lower levels of free IGF-1 in cells and tissues, indirectly reduce the risk of esophageal cancer (19). (3) Cellular Protective Effects: GLP-1 RAs may possess cytoprotective properties that promote cell survival and repair while suppressing apoptosis. This mechanism could help mitigate damage and mutations in esophageal epithelial cells, thereby reducing the likelihood of malignant transformation (20). (4) Enhanced Tissue Repair and Regeneration: GLP-1 RAs may enhance the repair and regenerative capacity of gastrointestinal epithelial cells, reducing the risk of cancer development due to prolonged inflammation and injury. (5) Inhibition of Cell Proliferation: Some studies suggest that GLP-1 RAs may inhibit the proliferation and growth of cancer cells through GLP-1 receptor-mediated pathways. This indicates that even in the presence of precancerous lesions, the use of GLP-1 RAs might suppress tumor progression (21).

In conclusion, treatment with GLP-1 RAs in adults with T2DM or obesity does not increase the risk of esophageal cancer, and there are probably no significant differences in risk across different GLP-1 RA types. These findings offer new therapeutic options for managing T2DM in clinical practice.

## Data Availability

The original contributions presented in the study are included in the article/**Supplementary Material**. Further inquiries can be directed to the corresponding authors.
