# Supplementary material for: A meta-analysis on the risk of esophageal cancer in type 2 diabetes patients treated with GLP-1 receptor agonists
Source: Front Endocrinol (Lausanne). 2025 Mar 20;16:1532587. doi: 10.3389/fendo.2025.1532587 (PMC11966957; doi:10.3389/fendo.2025.1532587)
Supplement: Supplementary file 1 [file DataSheet1.docx]

**Supplementary Appendix**

**A Meta-Analysis on the Risk of Esophageal Cancer in Type 2 Diabetes Patients Treated with GLP-1 Receptor Agonists**

Qi Wu^1,3,4, 7, #^, Yan Zeng^2,3,7, #^, Yong Liu^3,5, 6, #^, Fangyuan Teng^2,3^, Tiejun Zhou^1,4^,

Man Guo^2,3, *^, Zongzhe Jiang^2,3, *^, Yong Xu^2,3,7, *^

**Tables and figures of contents**

**Appendix 1**: Data sources and search strategies.

**Appendix 2.** PRISMA checklist.

**Appendix 3.** Eligibility criteria of included studies.

**Appendix 4.** Baseline characteristics of included RCTs.

**Appendix 5.** Classification and definition of different subgroups.

**Appendix 6.** Assessments of risks of bias of eligible studies.

**eFigure 1.** Overall risk of bias of all randomized clinical trials.

**eFigure 2.** Risks of esophageal neoplasms in patients with GLP-1 RAs treating for diabetes.

**Appendix 1. Data sources and search strategies**

**Table S1.** Search strategy of PubMed

| # | Searches |
| --- | --- |
| 1 | (Glucagon like peptide-1 receptor agonist OR GLP-1 receptor agonist [Title/Abstract])) OR (GLP1 OR GLP 1 OR GLP-1[Title/Abstract]) |
| 2 | (albiglutide OR syncria OR gsk716155 OR naliglutide OR tanzeum[Title/Abstract]) OR (dulaglutide OR ly 2189265 OR trulicity[Title/Abstract]) OR (exenatide OR bydureon OR Byetta OR ac 2993 OR ly 2148568[Title/Abstract]) OR (liraglutide OR NN 2211 OR victoza OR saxenda [Title/Abstract]) OR (lixisenatide OR lyxumia [Title/Abstract]) OR (semaglutide[Title/Abstract]) OR (taspoglutide[Title/Abstract]) |
| 3 | #1 OR #2 |
| 4 | ((Gastrointestinal Neoplasms OR Digestive System Neoplasms [MeSH Terms]) OR ((digestive OR gastrointestinal) AND (cancer OR carcinoma OR neoplas* OR tumor OR tumour OR malignan*)) [Title/Abstract]) |
| 5 | ((esophageal neoplasms [MeSH Terms])) OR ((esophag* OR oesophag*) AND (cancer OR carcinoma OR neoplas* OR tumor OR tumour OR malignan*) [Title/Abstract]) |
| 6 | #4 OR #5 |
| 7 | #3 AND #6 |
| 8 | (((random* [Title/Abstract])) OR (Randomized Controlled Trial [Title/Abstract])) OR (RCT or RCTs [Title/Abstract]) |
| 9 | #7 AND #8 |

**Table S2.** Search strategy of Embase

| # | Searches |
| --- | --- |
| 1 | ti, ab, kw: 'Glucagon like peptide-1 receptor agonis*' OR ti, ab, kw: 'GLP-1 receptor agonis*' OR ti, ab, kw: 'GLP1' OR ti, ab, kw: 'GLP 1' OR ti, ab, kw: 'GLP-1' |
| 2 | ti, ab, kw: albiglutide OR ti, ab, kw: dulaglutide OR ti, ab, kw: exenatide OR ti, ab, kw: liraglutide OR ti, ab, kw: lixisenatide OR ti, ab, kw: loxenatide OR ti, ab, kw: semaglutide OR ti, ab, kw: taspoglutide OR ti, ab, kw: tirzepatide |
| 3 | 'albiglutide'/exp OR 'dulaglutide'/exp OR ' exenatide '/exp OR ' liraglutide '/exp OR 'lixisenatide'/exp OR 'loxenatide'/exp OR 'semaglutide'/exp OR 'taspoglutide'/exp OR 'tirzepatide'/exp |
| 4 | #1 OR ~ #3 |
| 5 | 'gastrointestinal tumor'/exp OR ti, ab, kw: 'gastrointestinal tumor' OR 'digestive system tumor'/exp OR ti, ab, kw: 'digestive system tumor' |
| 6 | ('digestive system disease'/exp OR ti, ab, kw: 'digestive system disease') OR ('digestive system function disorder'/exp OR ti, ab, kw: 'digestive system function disorder') |
| 7 | 'malignant neoplasm'/exp OR 'ti, ab, kw: malignant neoplasm' OR 'carcinoma'/exp OR ti, ab, kw: carcinoma OR 'cancer'/exp OR ti, ab, kw: cancer OR 'neoplasm'/exp OR ti, ab, kw: neoplasm OR 'malignant'/exp OR ti, ab, kw: malignant OR 'benign'/exp OR ti, ab, kw: benign |
| 8 | #6 AND #7 |
| 9 | #5 OR #8 |
| 10 | 'esophagus tumor'/exp OR ti, ab, kw: 'esophagus tumor' OR 'esophagus cancer'/exp OR ti, ab, kw: 'esophagus cancer' OR 'esophagus carcinoma'/exp OR ti, ab, kw: 'esophagus carcinoma' OR 'esophagus malignant' OR (('esophagus'/exp OR esophagus) AND malignant) OR 'esophagus cancer' OR (esophagus AND ('cancer'/exp OR cancer)) |
| 11 | #9 OR #10 |
| 12 | #4 AND #11 |

**Table S3.** Search strategy of Scopus

| # | Searches |
| --- | --- |
| 1 | ((TITLE-ABS-KEY (glp1 OR "GLP 1" OR glp-1)) OR ((TITLE-ABS-KEY ("Glucagon like peptide-1 receptor agonist" OR "GLP-1 receptor agonist")) OR ((TITLE-ABS-KEY ("Glucagon like peptide-1")) |
| 2 | ((TITLE-ABS-KEY (semaglutide OR taspoglutide)) OR (TITLE-ABS-KEY ( lixisenatide OR lyxumia)) OR (TITLE-ABS-KEY (liraglutide OR nn 2211 OR victoza OR saxenda)) OR (TITLE-ABS-KEY (exenatide OR bydureon OR byetta OR ac 2993 OR ly 2148568)) OR (TITLE-ABS-KEY (dulaglutide OR ly 2189265 OR trulicity)) OR (TITLE-ABS-KEY (albiglutide OR syncria OR gsk716155 OR naliglutide OR tanzeum)) |
| 3 | #1 OR #2 |
| 4 | ((TITLE-ABS-KEY (cancer OR carcinoma OR neoplas* OR tumor OR tumour OR malignan*)) AND (TITLE-ABS-KEY (digestive OR gastrointestinal))) OR (TITLE-ABS-KEY ("gastrointestinal neoplasms" OR "digestive system neoplasms")) |
| 5 | (TITLE-ABS-KEY ("esophageal neoplasms")) OR ((TITLE-ABS-KEY (esophag* OR oesophag*)) AND (TITLE-ABS-KEY (cancer OR carcinoma OR neoplas* OR tumor OR tumour OR malignan*))) |
| 6 | #4 OR #5 |
| 7 | #3 AND #6 |
| 8 | #7 AND (LIMIT-TO (DOCTYPE, "ar") OR LIMIT-TO (DOCTYPE, "re")) AND (LIMIT-TO (EXACTKEYWORD, "Human")) AND (LIMIT-TO (SRCTYPE, "j")) |

**Table S4.** Search strategy of Web of Science: Science Citation Index Expanded

| # | Searches |
| --- | --- |
| 1 | TS=(GLP-1 receptor agonis*) OR TS=(Glucagon-like peptide-1 agonis*) |
| 2 | TS=(Glucagon like peptide-1 or Glucagon like peptide-1 receptor agonist OR GLP-1 receptor agonist or GLP1 OR GLP 1 OR GLP-1 or albiglutide OR syncria OR gsk716155 OR liraglutide OR tanzeum or dulaglutide OR ly 2189265 OR trulicity or exenatide OR bydureon OR Byetta OR ac 2993 OR ly 2148568 or liraglutide OR NN 2211 OR victoza OR saxenda or lixisenatide OR lyxumia or semaglutide or taspoglutide) |
| 3 | #1 OR #2 |
| 4 | TS=(random* OR randomized controlled trial OR RCT OR RCTs OR clinical trial) |
| 5 | #3 AND #4 |
| 6 | TS=(Gastrointestinal Neoplasms or Digestive System Neoplasms) |
| 7 | TS=((digestive OR gastrointestinal) AND (cancer OR carcinoma OR neoplas* OR tumor OR tumour OR malignan*)) |
| 8 | #6 OR #7 |
| 9 | TS=((esophag* OR oesophag*) AND (cancer OR carcinoma OR neoplas* OR tumor OR tumour OR malignan*)) OR TS=(esophageal neoplasms) |
| 10 | #8 OR #9 |
| 11 | #5 AND #10 |

**Table S5.** Search strategy of Cochrane Central Register of Controlled Trials (CENTRAL)

| # | Searches |
| --- | --- |
| 1 | ((GLP-1 receptor agonis*) OR (Glucagon-like peptide-1 agonis*)) ti,ab,kw |
| 2 | (Glucagon like peptide-1 or Glucagon like peptide-1 receptor agonist OR GLP-1 receptor agonist or GLP1 OR GLP 1 OR GLP-1 or albiglutide OR syncria OR gsk716155 OR liraglutide OR tanzeum or dulaglutide OR ly 2189265 OR trulicity or exenatide OR bydureon OR Byetta OR ac 2993 OR ly 2148568 or liraglutide OR NN 2211 OR victoza OR saxenda or lixisenatide OR lyxumia or semaglutide or taspoglutide) ti,ab,kw |
| 3 | #1 OR #2 |
| 4 | (Gastrointestinal Neoplasms or Digestive System Neoplasms) ti,ab,kw |
| 5 | ((digestive OR gastrointestinal) AND (cancer OR carcinoma OR neoplas* OR tumor OR tumour OR malignan*)) ti,ab,kw |
| 6 | #4 OR #5 |
| 7 | ((esophag* OR oesophag*) AND (cancer OR carcinoma OR neoplas* OR tumor OR tumour OR malignan*)) ti,ab,kw OR (esophageal neoplasms) ti,ab,kw |
| 8 | #6 OR #7 |
| 9 | #3 AND #8 |

**Table S6.** Search strategy of Clinical.gov

| # | Searches |
| --- | --- |
| 1 | Glucagon like peptide-1 receptor agonis* OR GLP-1 receptor agonis* OR GLP1 OR GLP-1 OR albiglutide OR dulaglutide OR exenatide OR liraglutide OR lixisenatide OR loxenatide OR semaglutide OR taspoglutide OR tirzepatide |
| 2 | Gastrointestinal Neoplasms OR Digestive System Neoplasms OR ((esophag* OR oesophag*) AND (cancer OR carcinoma OR neoplas* OR tumor OR tumour OR malignan*)) |
| 3 | #1 AND #2 |

**Appendix 2.** PRISMA checklist.

| **Section and Topic** | **Item #** | **Checklist item** | **Location where item is reported** |
| --- | --- | --- | --- |
| **TITLE** | | |  |
| Title | 1 | Identify the report as a systematic review. | 1 |
| ABSTRACT | | |  |
| Abstract | 2 | See the PRISMA 2020 for Abstracts checklist. | 1 |
| INTRODUCTION | | |  |
| Rationale | 3 | Describe the rationale for the review in the context of existing knowledge. | 2 |
| Objectives | 4 | Provide an explicit statement of the objective(s) or question(s) the review addresses. | 2 |
| METHODS | | |  |
| Eligibility criteria | 5 | Specify the inclusion and exclusion criteria for the review and how studies were grouped for the syntheses. | 2 |
| Information sources | 6 | Specify all databases, registers, websites, organisations, reference lists and other sources searched or consulted to identify studies. Specify the date when each source was last searched or consulted. | 2 |
| Search strategy | 7 | Present the full search strategies for all databases, registers and websites, including any filters and limits used. | 2 |
| Selection process | 8 | Specify the methods used to decide whether a study met the inclusion criteria of the review, including how many reviewers screened each record and each report retrieved, whether they worked independently, and if applicable, details of automation tools used in the process. | 2 |
| Data collection process | 9 | Specify the methods used to collect data from reports, including how many reviewers collected data from each report, whether they worked independently, any processes for obtaining or confirming data from study investigators, and if applicable, details of automation tools used in the process. | 2 |
| Data items | 10a | List and define all outcomes for which data were sought. Specify whether all results that were compatible with each outcome domain in each study were sought (e.g. for all measures, time points, analyses), and if not, the methods used to decide which results to collect. | 2 |
|  | 10b | List and define all other variables for which data were sought (e.g. participant and intervention characteristics, funding sources). Describe any assumptions made about any missing or unclear information. | 2 |
| Study risk of bias assessment | 11 | Specify the methods used to assess risk of bias in the included studies, including details of the tool(s) used, how many reviewers assessed each study and whether they worked independently, and if applicable, details of automation tools used in the process. | 3 |
| Effect measures | 12 | Specify for each outcome the effect measure(s) (e.g. risk ratio, mean difference) used in the synthesis or presentation of results. | 3 |
| Synthesis methods | 13a | Describe the processes used to decide which studies were eligible for each synthesis (e.g. tabulating the study intervention characteristics and comparing against the planned groups for each synthesis (item #5)). | 3 |
|  | 13b | Describe any methods required to prepare the data for presentation or synthesis, such as handling of missing summary statistics, or data conversions. | 3 |
|  | 13c | Describe any methods used to tabulate or visually display results of individual studies and syntheses. | 3 |
|  | 13d | Describe any methods used to synthesize results and provide a rationale for the choice(s). If meta-analysis was performed, describe the model(s), method(s) to identify the presence and extent of statistical heterogeneity, and software package(s) used. | 3 |
|  | 13e | Describe any methods used to explore possible causes of heterogeneity among study results (e.g. subgroup analysis, meta-regression). | 3 |
|  | 13f | Describe any sensitivity analyses conducted to assess robustness of the synthesized results. | 3 |
| Reporting bias assessment | 14 | Describe any methods used to assess risk of bias due to missing results in a synthesis (arising from reporting biases). | 3 |
| Certainty assessment | 15 | Describe any methods used to assess certainty (or confidence) in the body of evidence for an outcome. | 3 |
| **RESULTS** | | |  |
| Study selection | 16a | Describe the results of the search and selection process, from the number of records identified in the search to the number of studies included in the review, ideally using a flow diagram. | 3 |
|  | 16b | Cite studies that might appear to meet the inclusion criteria, but which were excluded, and explain why they were excluded. | 3 |
| Study characteristics | 17 | Cite each included study and present its characteristics. | 3 |
| Risk of bias in studies | 18 | Present assessments of risk of bias for each included study. | 3 |
| Results of individual studies | 19 | For all outcomes, present, for each study: (a) summary statistics for each group (where appropriate) and (b) an effect estimate and its precision (e.g. confidence/credible interval), ideally using structured tables or plots. | 3 |
| Results of syntheses | 20a | For each synthesis, briefly summarise the characteristics and risk of bias among contributing studies. | 3 |
|  | 20b | Present results of all statistical syntheses conducted. If meta-analysis was done, present for each the summary estimate and its precision (e.g. confidence/credible interval) and measures of statistical heterogeneity. If comparing groups, describe the direction of the effect. | 3 |
|  | 20c | Present results of all investigations of possible causes of heterogeneity among study results. | 3 |
|  | 20d | Present results of all sensitivity analyses conducted to assess the robustness of the synthesized results. | 3 |
| Reporting biases | 21 | Present assessments of risk of bias due to missing results (arising from reporting biases) for each synthesis assessed. | 3 |
| Certainty of evidence | 22 | Present assessments of certainty (or confidence) in the body of evidence for each outcome assessed. | 3 |
| DISCUSSION | | |  |
| Discussion | 23a | Provide a general interpretation of the results in the context of other evidence. | 3 |
|  | 23b | Discuss any limitations of the evidence included in the review. | 3-4 |
|  | 23c | Discuss any limitations of the review processes used. | 3-4 |
|  | 23d | Discuss implications of the results for practice, policy, and future research. | 4 |
| OTHER INFORMATION | | |  |
| Registration and protocol | 24a | Provide registration information for the review, including register name and registration number, or state that the review was not registered. | 2 |
|  | 24b | Indicate where the review protocol can be accessed, or state that a protocol was not prepared. | 2 |
|  | 24c | Describe and explain any amendments to information provided at registration or in the protocol. | 2 |
| Support | 25 | Describe sources of financial or non-financial support for the review, and the role of the funders or sponsors in the review. | 7 |
| Competing interests | 26 | Declare any competing interests of review authors. | 7 |
| Availability of data, code and other materials | 27 | Report which of the following are publicly available and where they can be found: template data collection forms; data extracted from included studies; data used for all analyses; analytic code; any other materials used in the review. | 7 |

*From:* Page MJ, McKenzie JE, Bossuyt PM, Boutron I, Hoffmann TC, Mulrow CD, et al. The PRISMA 2020 statement: an updated guideline for reporting systematic reviews. BMJ 2021;372:n71. doi: 10.1136/bmj.n71

For more information, visit: <http://www.prisma-statement.org/>

**Appendix 3.** Eligibility criteria of included studies.

|  | Inclusion criteria | Exclusion criteria |
| --- | --- | --- |
| Participants | Adults (≥ 18 or 20 years old); with type 2 diabetes, obesity or overweight. | Participants < 18 years old or receiving GLP-1 RAs treatments before the trials. |
| Intervention | Any types, any dose and frequency of GLP-1 RAs; GLP-1 RAs used as monotherapy or add-on treatments to other interventions or hypoglycemic agents with minimum intervention ≥ 52 weeks | Co-formulation of fixed-dose combinations of GLP-1 RAs other antidiabetic drugs (e.g., IDegLira is a fixedratio combination of insulin degludec and liraglutide). |
| Comparators | Placebo, non-GLP-1 RAs medications or other interventions (e.g., dietary management, physical exercise). | Comparison of different type, frequency, or dose of GLP-1 RAs. Comparison of different administration methods of one type of GLP-1 RAs (subcutaneous semaglutide vs. oral semaglutide) without a placebo arm. |
| Outcomes | Reporting the outcomes in one or more of: gastrointestinal neoplasms, digestive system neoplasm, esophageal neoplasms. | No primary or secondary outcome data available. |
| Study design | Randomized controlled clinical trials (RCTs). | Reviews or case reports, conference abstract, and expert opinion; uncompleted or withdrawn studies |

Note: GLP-1 RAs: glucagon-like peptide 1 receptor agonists.

**Appendix 4.** Baseline characteristics of included RCTs.

| Study | Inclusion Period | Follow up | Randomised treatments | duration of diabetes or obesity, (mean, SD), years | Total cases | Completed cases | Age (mean, SD), years | No. (%) of male | Wight ( mean, SD), kg | BMI (mean, SD), kg/m2 | Waist circumference (cm) | FPG (mmol/l) | HbA1c (mean, SD), % | No. (%) of White | No. (%) of Black or African American | No. (%) of Asian | No. (%) of Others |
| --- | --- | --- | --- | --- | --- | --- | --- | --- | --- | --- | --- | --- | --- | --- | --- | --- | --- |
| Sponsor:  GlaxoSmithKline | Jul 1, 2015 to Nov 24, 2016 | 56 days | Albiglutide 30, 50 mg QW +glargine, SC; Lispro + glargine | 14.1 (8.6) 14.2 (8.9) | 4731 4732 | 4620 4578 | 64.1 (8.71) 64.2 (8.65) | 3304 (69.8) 3265 (69.0) | NR | 32.3 (5.9) 32.3 (5.9) | NR | NR | 8.8 (1.5) 8.7 (1.5) | 4006 (84.7) 4024 (85.0) | 121 (2.6) 118 (2.5) | 325 (6.9) 242 (5.1) | 279 (5.9) 348 (7.4) |
| M. Diamant, 2012 | May 13, 2008 to Jan 30, 2012 | > 2.5 years | Exenatide 2 mg QW, SC; Insulin glargine: 10 IU/day | 8.0 (6.0) 7.8 (6.0) | 233 223 | 173 173 | 58.0 (10.0) 58.0 (9.0) | 120 (52.0) 123 (55.0) | 91.2 (18.6) 90.6 (16.4) | 32.0 (5.0) 32.0 (5.0) | 106.7 (0.8) 107.2 (0.8) | 9.9 (2.5) 9.7 (2.7) | 8.3 (1.1) 8.3 (1.0) | 190 (82.0) 189 (85.0) | 2 (1.0) | 13 (6.0) 14 (6.0) | 28 (12.0)* 19 (9.0) |
| M. Diamant, 2014 | May 13, 2008 to Jan 30, 2012 | 3 years | Exenatide 2 mg QW, SC; Insulin glargine: 10 IU/day | 8.0 (6.0) 7.8 (6.0) | 233 223 | 140 147 | 58.0 (10.0) 58.0 (9.0) | 120 (52.0) 123 (55.0) | 91.2 (18.6) 90.6 (16.4) | 32.0 (5.0) 32.0 (5.0) | 106.7 (0.8) 107.2 (0.8) | 9.9 (2.5) 9.7 (2.7) | 8.3 (1.1) 8.3 (1.0) | 190 (82.0) 189 (85.0) | 1 (0.0) | 13 (6.0) 14 (6.0) | 28 (12.0)* 19 (9.0) |
| K. Kaku, 2018 | Aug 4, 2014 to Feb 27, 2016 | 5 weeks | Semaglutide 0.5 mg QW, SC;  Semaglutide 1.0 mg QW, SC;  Additional OAD | 8.1 (6.0) 9.4 (6.5) 9.3 (7.0) | 239 241 121 | 233 231 115 | 58.0 (10.6) 58.7 (10.2) 59.2 (10.1) | 166 (69.5) 174 (72.2) 90 (74.2) | 71.0 (15.4) 71.7 (15.9) 72.2 (14.9) | 26.2 (4.8) 26.4 (4.7) 26.7 (4.6) | NR | 8.9 (1.9) 8.9 (2.1) 9.0 (1.9) | 8.0 (0.9) 8.1 (1.0) 8.1 (0.9) | 0 (0.0) | 0 (0.0) | 239 (100.0) 241 (100.0) 121 (100.0) | 0 (0.0) |
| M. Kellerer, 2022 | Oct 2018 to Oct 2019 | 5 weeks | Semaglutide 1.0 mg QW, SC; Insulin | 13.4 (6.8) 13.4 (6.5) | 874 874 | 850 831 | 60.8 (9.4) 61.5 (9.5) | 445 (50.9) 449 (51.4) | 87.6 (18.1) 88.1 (18.4) | 31.4 (5.5) 31.7 (5.5) | NR | NR | 8.6 (0.7) 8.5 (0.7) | 674 (77.1) 691 (79.1) | 21 (2.4) 14 (1.6) | 176 (20.1) 166 (19.0) | 3 (0.3) 3 (0.3) |
| F. K. Knop, 2023 | Sept 13, 2021 to Nov 22, 2021 | 7 weeks | Semaglutide 50 mg QD, PO; Placebo | NR | 334 333 | 320 307 | 49.0 (13.0) 50.0 (12.0) | 87 (26.0) 95 (29.0) | 104.5 (22.0) 106.2 (22.3) | 37.3 (6.3) 37.7 (6.8) | 112.6 (13.6) 114.5 (15.4) | 5.4 (0.7) 5.5 (0.5) | 5.6 (0.3) 5.6 (0.3) | 246 (74.0) 248 (74.0) | 21 (6.0) 22 (7.0) | 36 (11.0) 36 (11.0) | 31 (9.0) 27 (8.0) |

Note: *Indicating that the population is mainly Hispanic; Round the data to one decimal place. Abbreviations: Ref., references; NO., number of total participants; BMI, body mass index; FPG, fasting plasma glucose; HbA1c, glycosylated haemoglobin; SC, subcutaneous injections; PO, oral intake; NR, not report; NA, not available.

**Appendix 5.** Classification and definition of different subgroups.

- 1. Baseline mean age: ≤60 or >60 years.
  2. Baseline mean BMI: (1) Overweight: 25 ≤ BMI < 30; (2) Obesity: BMI ≥ 30.
  3. Treatment durations: (1) ≤52 weeks; (2) 52-104 weeks; (3) >104 weeks.
  4. Type of control: placebo or another oral antidiabetic drug (Active comparator);
  5. Indication for treatment: type 2 diabetes or obesity.
  6. The different doses of each GLP-1 RA:

1. High doses of GLP-1 RAs were defined as equal to or greater than: albiglutide, 50 mg once weekly; exenatide, 10 μg twice daily; dulaglutide, 1.5 mg once weekly; liraglutide, 1.8 mg once daily; lixisenatide, 20 μg once daily; subcutaneous semaglutide, 1.0 mg once weekly; and oral semaglutide, 7 mg or 14 mg once daily.
2. Low doses were defined as: albiglutide, 30 mg (<50 mg) once weekly; exenatide, 5 μg twice daily; dulaglutide, 0.75 mg (<1.5 mg) once weekly; liraglutide, 0.9 to 1.2 mg (<1.8 mg) once daily; lixisenatide, 10 μg once daily; subcutaneous semaglutide, 0.5 mg (<1.0 mg) once weekly; and oral semaglutide, 3.0 mg (<7.0 mg) once daily.

**Appendix 6.** Assessments of risks of bias of eligible studies.

**Version 2 of the Cochrane risk-of-bias tool for assessing risk of bias of randomized clinical trials.**

| Study | Cohort name or NCT | Study-type | Randomisation | Deviations from intended interventions | Missing outcome data | Measurement of the outcome | Selection of the reported result | Overall risk of bias |
| --- | --- | --- | --- | --- | --- | --- | --- | --- |
| Sponsor: GlaxoSmithKline | NCT02465515 (Harmony Outcomes) | double-blind, randomized, placebo-controlled, event-driven trial | Low | Low | Low | Low | Low | **Low risk** |
| M. Diamant, 2012 | NCT00641056 (DURATION-3) | multicenter, open-label, randomized, two-arm, parallel, comparator-controlled trial | Low | Low | Some concerns | Low | Low | **Some concerns** |
| M. Diamant, 2014 | NCT00641056 (DURATION-3) | multicenter, open-label, randomized, two-arm, parallel, comparator-controlled trial | Low | Low | Some concerns | Low | Low | **Some concerns** |
| K. Kaku, 2018 | NCT02207374 | phase 3 randomized, open-label, active-controlled, parallel-group, multicenter trial | Low | Low | Low | Low | Low | **Low risk** |
| M. Kellerer, 2022 | NCT03689374  (SUSTAIN 11) | randomized, parallel, open-label, multinational, phase 3b trial | Low | Low | Low | Low | Low | **Low risk** |
| F. K. Knop, 2023 | NCT05035095  (OASIS 1) | randomized, double-blind, placebo-controlled, phase 3 trial | Low | Low | Low | Low | Low | **Low risk** |

Note: Each domain of risk was assigned “Low” for low risk, “Moderate” for moderate risk, and “Some concerns” for certain risks or issues present that not sufficient to fully impact the study's conclusions.

**eFigure 1.** Overall risk of bias of all randomized clinical trials.

**Overall risk of bias presented as percentage of each risk of bias item across all included studies.**

**eFigure 2.** Risks of esophageal neoplasms in patients with GLP-1 RAs treating for diabetes.

**
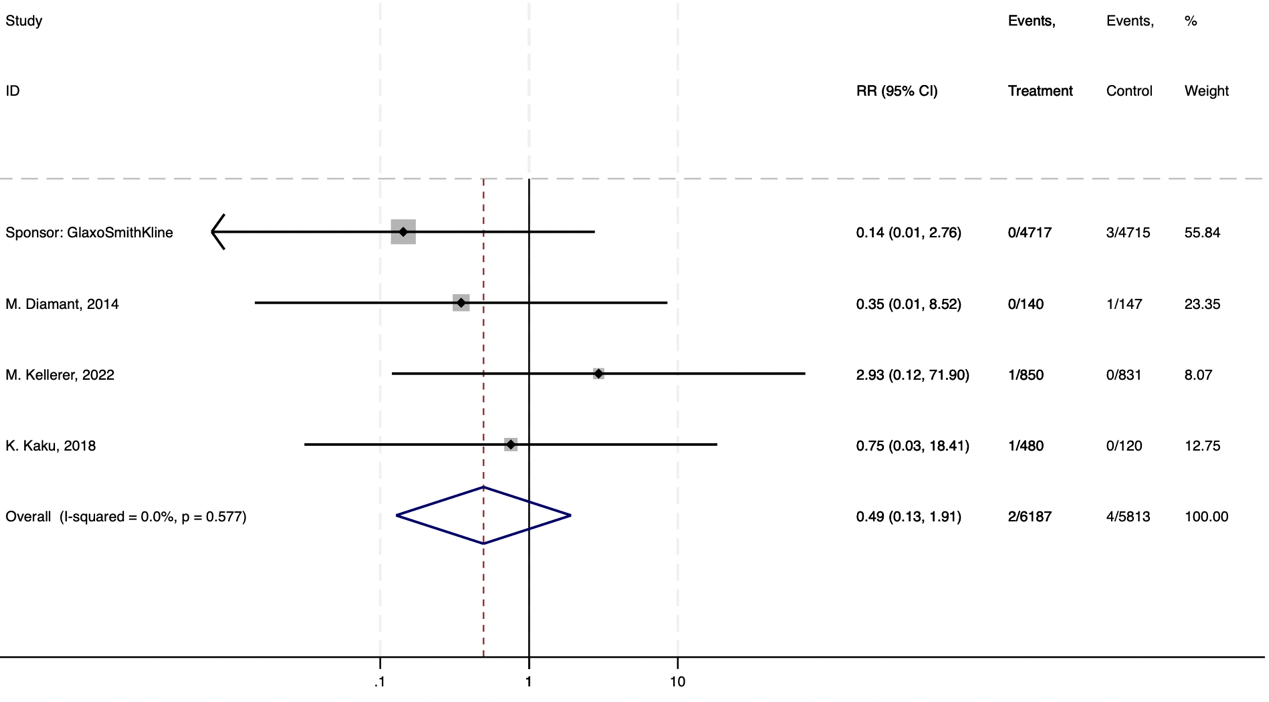
**
